# Addressing treatment contamination in the design and analysis of trials of complex interventions: early results from a systematic review of mental health trials affected by contamination

**DOI:** 10.1186/1745-6215-16-S2-P7

**Published:** 2015-11-16

**Authors:** Nicholas Magill, Khalida Ismail, Paul McCrone, Sabine Landau

**Affiliations:** 1 King's College London, London, UK

## 

In mental health trials there is concern that the control treatment (therapy) might be contaminated due to it being delivered by a clinician (therapist) who has been trained in the active intervention or has been in contact with a professional who has received this training. It is often suggested by investigators, reviewers or funders of clinical trial proposals that cluster randomisation, with clusters defined at the level at which treatment contamination occurs (e.g. therapists), should be used to prevent contamination.

The overall aim of the research is to investigate whether it is more efficient to avoid contamination through the use of cluster randomisation or to use individual randomisation and account for it in the analysis. The prediction is that cluster randomisation will only be favoured under high levels of contamination, modest intraclass correlation coefficients and small cluster sizes.

A systematic literature review is being conducted to assess the processes by which contamination occurs in mental health trials, for example crossover of clinical staff between trial arms or communication between staff trained in the new and old interventions. The review is also identifying the approaches (apart from cluster randomisation) that trialists use to address this problem. The final purpose of the review is to obtain estimates of parameters that would be needed to simulate a cluster randomized trial or a contamination process.

